# Mechanical and Ecological Properties of CO_2_ Curing Magnesium Slag Concrete

**DOI:** 10.3390/ma18010109

**Published:** 2024-12-30

**Authors:** Lu Zhang, Yilong Zhang, Fan Zhang, Haonan Liang, Ditao Niu, Hui Li

**Affiliations:** 1 College of Materials Science and Engineering, Xi’an University of Architecture and Technology, Xi’an 710055, China; zhanglu@live.xauat.edu.cn (L.Z.); zhang003011@163.com (Y.Z.); liang2160644661@163.com (H.L.); 2 School of Architecture and Construction, Xi’an University of Architecture and Technology, Xi’an 710055, China; zz7410666@163.com; 3 State Key Laboratory of Green Building in Western China, Xi’an University of Architecture and Technology, Xi’an 710055, China; niuditao@163.com; 4 Ecological Cement Engineering Research Center of Ministry of Education, Xi’an 710055, China; 5 Shaanxi Ecological Cement and Concrete Engineering Technology Research Center, Xi’an 710055, China

**Keywords:** magnesium slag concrete, carbon curing, carbon fixation, hydration reaction, mechanical properties

## Abstract

Magnesium slag is a by-product of the magnesium industry. As an auxiliary cementitious material incorporated into concrete, it can make full use of waste resources and has a certain potential for hydration and carbonation. To improve the mechanical properties of the concrete, the influence mechanism and strengthening mechanism of the carbon curing method on mechanical properties of magnesium slag concrete were investigated. The effects of different magnesium slag content and water-cement ratio on mechanical properties and ecological properties of carbon cured magnesium slag concrete were analyzed. Based on the phase composition and thermogravimetric composition of magnesium slag concrete, the carbonation mechanism of magnesium slag was revealed. The mechanical properties models of magnesium slag concrete with different carbon curing were constructed. The study shows that with the increase of the magnesium slag, the mechanical properties of carbon curing concrete first increase and then decrease. The optimum mechanical properties of concrete are 30% magnesium slag, and the compressive strength reaches 42.3 MPa. The content of magnesium slag increased from 0% to 60%, and the carbon fixation content was 14.60%, 11.87%, 11.69%, 16.90%, 19.80%, 14.78%, and 13.09%, respectively. With the increase of magnesium slag content, the content and grain size of magnesium oxide in concrete increase, which leads to more micro-bumps and depressions on the surface of the concrete structure. The hydration reaction and carbonation reaction of gelled materials are affected by magnesium ions, resulting in changes in the morphology and crystal structure of CaCO_3_ and MgCO_3_ reactants.

## 1. Introduction

Magnesium slag (MS) is a by-product of smelting magnesium, and each ton of metal magnesium smelting will produce 6 to 10 tons of MS [1]. At present, the resource utilization of MS is not mature, and MS is piled up at random, resulting in the waste of land resources and environmental pollution [2]. With the attention of various countries to the environment, under the background of the “double-carbon plan”, efficient resource utilization of MS has become a research hotspot in the field of solid waste resource utilization.

At present, the research on MS material is relatively dispersed, and it has been involved in the preparation of clinker [3,4,5,6,7], fiber [8], ceramics [9], and agriculture [10]. However, the above application approach has the disadvantage of affecting its own process quality or being unable to use MS on a large scale, which cannot really solve the problem of a large amount of MS [11]. It is found that the internal composition of MS is mainly dicalcium silicate (C_2_S) [12], which has good potential hydration activity and carbonation activity. Therefore, it has excellent application potential in the field of building materials and can obtain good properties to meet the needs of engineering by grinding, chemical excitation, and carbonation curing. Sun Rui et al. ground MS to obtain a larger reaction specific surface area, which greatly improves hydration activity, and the surface shape of MS particles to enhances sphericity [13,14]. Other scholars used MS and a variety of materials (slag [15], cement [15], quicklime [16], NaOH [16], water glass [17], and composite stimulating agent [18]) to promote the excitation of magnesium residue through the high alkaline environment, which enhanced the performance of MS gelling materials.

Magnesium slag concrete (MSC) is a building material with wide application feasibility. The main chemical property of MSC is the chemical reaction of cement, MS, and water to produce hydrate and hardening products [19]. The hardening time of MSC is shorter than that of ordinary concrete, but the hardening rate of MSC will be slower in high temperature and humid environments. The hydration heat of MSC is high, so it is necessary to control the temperature during construction. The expansion of MSC in the process of hydration should be controlled during construction [20]. The main mineral components in MSC include magnesium oxide, calcium silicate, hydration calcium silicate, etc., which can improve the corrosion resistance of concrete [21]. MS is a solid waste that needs to be consumed urgently, and its chemical composition is closer to cement than fly ash, slag, etc., so it can be incorporated on a large scale without affecting the performance of the prepared concrete. In addition, the high calcium properties of MS make it an ideal raw material for carbonation, which can give concrete strength compensation after carbonation curing. Therefore, MS as an auxiliary gelling material has more ecological advantages. When MS is utilized as a partial replacement for cement, it may necessitate more water than common solid wastes, such as fly ash [22] and silica fume [23]. Furthermore, due to its similarity to the calcium silicate phase of steel slag [24] and its high carbonation activity, its carbonation potential is huge.

In recent years, carbonation curing has attracted significant attention as a new curing method for cementable materials. Its principle is to quickly generate a large amount of CaCO_3_ and active silica gel through the direct reaction between CO_2_ and minerals, which improves the performance of the cementitious material [25,26]. In addition, carbonation maintenance can absorb the CO_2_ produced by industry. It has reached the purpose of reducing carbon emissions, which is consistent with the current reform requirements of the construction industry. Hao Y. et al. used carbonation curing to treat MS mortar [27,28,29], which greatly increased the compressive strength of MS mortar, and the matrix substrate became denser [30]. The above research shows that carbonation curing can improve the mechanical properties of MS cementing material, but the carbonation law of MS in concrete and the durability of magnesium carbide slag concrete have not been proved, which cannot guide the practical engineering application [31]. Therefore, it is necessary to conduct more in-depth research in this field to promote the application of MS in the field of building materials.

In this study, the influence of carbon curing on the mechanical and ecological properties of MSC was studied by changing the MS content and water-cement ratio. The X-ray diffraction (XRD) and thermogravimetric differential thermal synthesis thermal analyzer (TG-DTG) were used to study the carbonation reaction mechanism and the change law of carbon curing MSC. Based on the compressive and flexural strength of MSC, a mechanical property model of carbon cured MSC was established.

## 2. Experimental Raw Materials and Test Process

### 2.1. Experimental Raw Materials

The cement used in the experiment was P.O 42.5R grade ordinary Portland cement with an apparent density of 3.0 g/cm^3^. The physicochemical properties are shown in Table 1 and Table 2.

**Figure 1:**
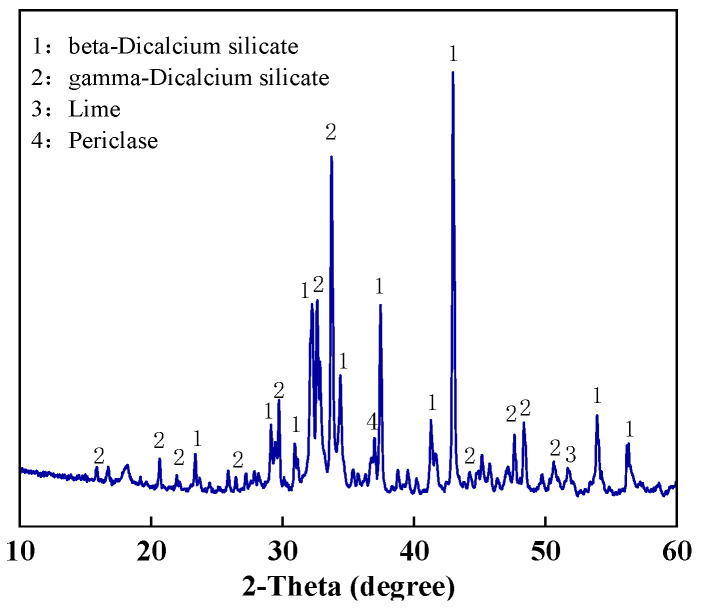
MS raw material composition.

The MS is dried at 105 °C for 3 h and then placed into the ball mill for 60 min. The processed MS is one of the main raw materials of the concrete cementing material. The chemical composition was analyzed using X-ray fluorescence spectroscopy (XRF) and the results are shown in Table 2. The main chemical components of MS are CaO and SiO_2_, and the content is close to 90%. In addition, it also contains a small amount of MgO, Fe_2_O_3_, Al_2_O_3_, etc. The phase composition was analyzed using X-ray diffraction (XRD), as shown in Figure 1. The characteristic peaks of *β* and *γ* dicalcium silicate in MS indicate that Ca and Si exist mainly as calcium silicate. There is also a small amount of free calcium oxide and calcite in the MS.

The fine aggregate used in the experiment is river sand with a fineness modulus of 2.8, mud content of 0.3%, apparent density of 2.65 g/cm^3^, and bulk density of 1.44 g/cm^3^. The coarse aggregate is gravel with a particle size of 5~20 mm, with an apparent density of 2.65 g/cm^3^ and a bulk density of 1.45 g/cm^3^. Polycarboxylic acid superplasticizer with 40% solid content, 30% water reduction rate, 6.5% alkali content, and 0.18% chloride ion content were used in the test.

### 2.2. Preparation of Carbon Curing MSC

The mix ratio of MSC is designed, and the water-cement ratio and the MS content are the changing factors. The MS content is 0%, 10%, 20%, 30%, 40%, 50%, and 60% and the corresponding numbers are I0, I10, I20, I30, I40, I50, I60. When the amount of MS is 30%, the MSC-I30, MSC-II30 and MSC-III30 are designed, and the corresponding to water-cement ratios are 0.59, 0.47, and 0.39, respectively. The combination of MSC is shown in Table 3.

**Figure 2:**
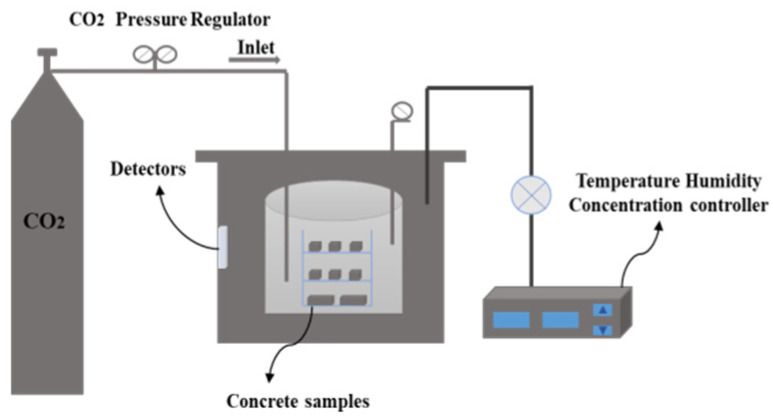
Schematic diagram of the carbonation reaction device.

In the experiment, an HJS-60 concrete double horizontal shaft mixer (Liansi Kaite (Hebei) Instrument Co., Ltd., Cangzhou, China) is used to mix concrete. First, add sand and cementing material (including cement and MS) to stir for 5 min, then slowly and evenly add water with water reducing agent to stir for 5 min. After the slurry is fully mixed, put it into 100 mm × 100 mm × 100 mm and 100 mm × 100 mm × 400 mm concrete molds for 24 h. The concrete test block is demolded and put into the carbonation curing device for curing for 1 d, and then taken out for standard curing to the corresponding age. The carbonation curing system is CO_2_ concentration of 25%, temperature of 30 °C, and humidity of 70%. The carbonation reaction device is shown in Figure 2.

### 2.3. Determination of MSC Performance

The mechanical properties of MSC with different mix ratios were tested, including compressive strength and flexural strength. The X-ray diffractometer (Rigaku Corporation, Tokyo, Japan) was used to analyze the phase composition inside the specimen. The synchronous thermal analysis of MSC was carried out to determine the carbon sequestration content (CSC) of MSC. The CSC in concrete is calculated by using the mass change of 550°C–900°C, as shown in Equation (1).

(1) $$\mathrm{CSC}=\frac{{\mathrm{Material}\;\mathrm{mass}\;\mathrm{at}\;550}^{\circ }-{\mathrm{Material}\;\mathrm{mass}\;\mathrm{at}\;900}^{\circ }}{\mathrm{Total}\;\mathrm{material}\;\mathrm{mass}}$$

## 3. Mechanical Properties of Carbon Curing MSC

### 3.1. Compressive Strength of MSC

The influence of the MS content on the compressive strength of the MSC at different ages (3 d, 7 d, 14 d, and 28 d) was studied. The compressive strength of MSC with different water-cement ratios (0.39, 0.47, 0.59) was studied.

#### 3.1.1. Influence of MS Content on Compressive Strength

**Figure 3:**
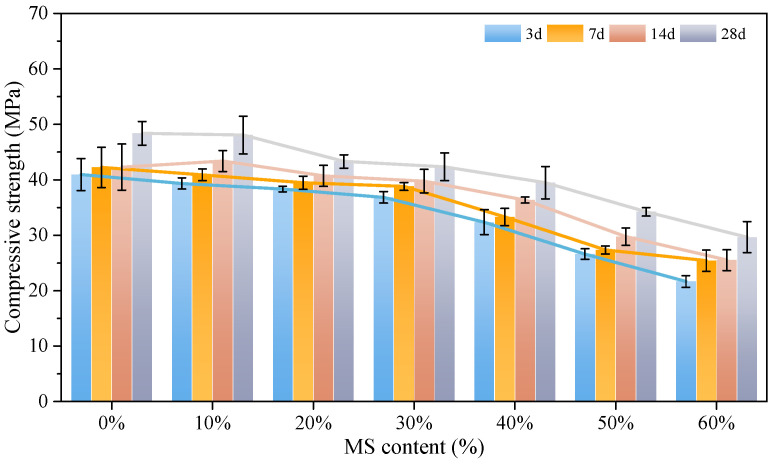
Compressive strength of carbon curing MSC with different MS content.

The compressive strength of concrete was analyzed with different MS contents, which were 0%, 10%, 20%, 30%, 40%, 50%, and 60%. The change curve of the compressive strength with age is shown in Figure 3.

It is concluded from Figure 3 that with the increase of MS content, the strength of MSC shows an obvious decreasing trend. When the MS content is 0~60% for 3 d, the compressive strengths of MSC were 40.9, 39.3, 38.3, 36.8, 32.4, 26.6, and 21.7 MPa, respectively. Compared with the concrete without MS, the compressive strength reduction rates were 1.47%, 3.91%, 10.02%, 20.78%, 34.96%, and 46.94%, respectively. When the MS content is 0~60% for 7 d, the compressive strengths of MSC were 42.2, 40.9, 39.3, 38.8, 33.3, 27.4, and 25.4 MPa, respectively. Compared with the concrete without MS, the compressive strength reduction rates were 3.08%, 6.87%, 8.06%, 21.09%, 35.07%, and 39.81%, respectively. When the MS content is 0~60% for 14 d, the compressive strengths of MSC were 42.3, 43.4, 40.7, 39.8, 36.3, 29.8, and 25.5 MPa, respectively. Compared with the concrete without MS, the compressive strength reduction rates were −2.60%, 3.78%, 5.91%, 14.18%, 29.55%, and 39.72%, respectively. When the MS content is 0~60% for 28 d, the compressive strengths of MSC were 48.34, 48.1, 43.3, 42.3, 39.5, 34.2, and 29.7 MPa, respectively. Compared with the concrete without MS, the compressive strength reduction rates were 0.50%, 10.43%, 12.49%, 18.29%, 29.25%, and 38.56%, respectively.

In this study, we set a strength reduction of less than 15% to meet the strength requirement. The results show that the design strength can be satisfied when the content of MS is less than or equal to 30%. When the MS content is higher than 30%, the compressive strength of concrete decreases rapidly. When the MS content is higher than 60%, the concrete can only reach 52.6% of the concrete without MS in 3 d. With the increase of curing age, the disadvantage of compressive strength of multi-MS content MSC is more obvious. After 28 d, the compressive strength of concrete is only 29 MPa, which fails to meet the designed strength requirements. The reason for this phenomenon may be due to the low hydration activity of MS [32], which can lead to the lack of hydration products in the concrete. This can cause significant porosity in the concrete, and the porosity of the pore structure is not good for the matrix to provide good mutual adhesion, which causes the mechanical properties to decrease.

#### 3.1.2. Influence of Water-Cement Ratio on Compressive Strength

**Figure 4:**
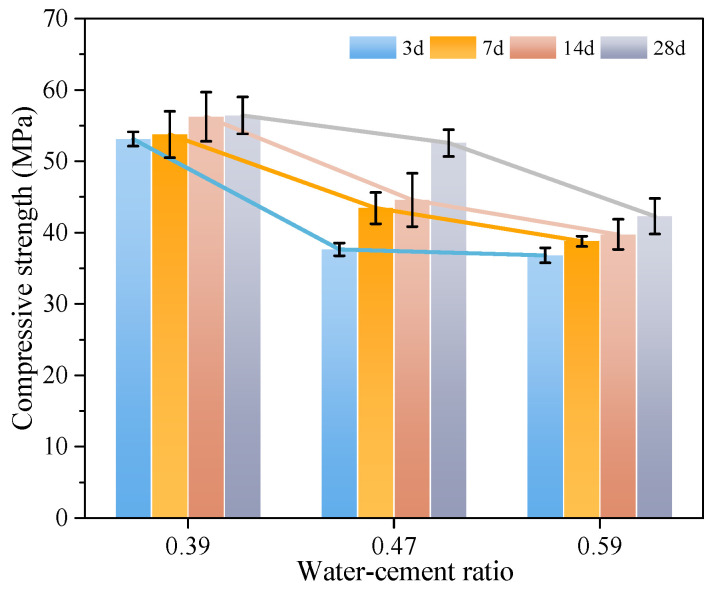
Compressive strength of carbon curing MSC with different water-cement ratio.

To study the influence of different water-cement ratios on the compressive strength of MSC, the compressive strength of MSC with curing age under water-cement ratios of 0.59, 0.47, and 0.39 was analyzed. The compressive strength of concrete with different water-cement ratios is shown in Figure 4.

It is concluded from Figure 4 that with the increase of water-cement ratio, the strength of MSC shows an obvious decreasing trend. When the water-cement ratios are 0.39, 0.47, and 0.59 for 3 d, the compressive strengths of MSC were 53.1, 37.7, and 36.8 MPa, respectively. Compared with the MSC of 0.39 water-cement ratio, the compressive strengths were reduced by 15.4 and 16.3 MPa, and the reduction rates were 29.0% and 30.70%, respectively. When the water-cement ratios are 0.39, 0.47, and 0.59 for 7 d, the compressive strengths of MSC were 53.8, 43.4, and 38.8 MPa, respectively. Compared with the MSC of 0.39 water-cement ratio, the compressive strengths were reduced by 10.4 and 15.0 MPa, and the reduction rates were 19.33% and 27.88%, respectively. When the water-cement ratios are 0.39, 0.47, and 0.59 for 14 d, the compressive strengths of MSC were 56.3, 44.6, and 39.8 MPa, respectively. Compared with the MSC of 0.39 water-cement ratio, the compressive strengths were reduced by 11.7 and 16.5 MPa, and the reduction rates were 20.78% and 29.31%, respectively. When the water-cement ratios are 0.39, 0.47, and 0.59 for 28 d, the compressive strengths of MSC were 56.4, 52.6, and 42.3 MPa, respectively. Compared with the MSC of 0.39 water-cement ratio, the compressive strengths were reduced by 3.8 and 14.1 MPa, and the reduction rates were 6.74% and 25%, respectively.

### 3.2. Flexural Strength of MSC

#### 3.2.1. Influence of MS Content on Flexural Strength

**Figure 5:**
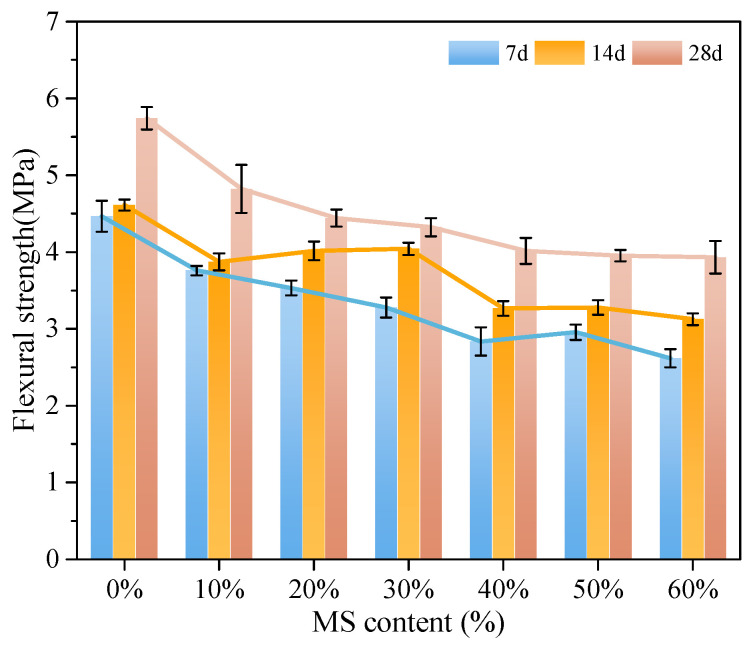
Flexural strength of carbon curing MSC with different MS content.

The flexural strength of MSC was analyzed with different MS contents, which were 0%, 10%, 20%, 30%, 40%, 50%, and 60%. The change curve of the flexural strength with curing age is shown in Figure 5.

It is concluded from Figure 5 that the flexural strength of carbon curing MSC with different MS content is affected by different MS content. With the increase of MS content, the flexural strength of MSC shows an obvious decreasing trend.

When the content of MS is less than 30%, the flexural strength decreases rapidly; when the content of MS is more than 30%, the decline slows down and becomes stable. When the MS content is 0~60% for 7 d, the flexural strengths of MSC were 4.5, 3.78, 3.53, 3.28, 2.8, 2.96, and 2.68 MPa, respectively. Compared with the concrete without MS, the flexural strength reduction rates were 16.0%, 21.56%, 27.11%, 37.78%, 34.22%, and 40.44%, respectively. When the MS content is 0~60% for 14 d, the flexural strengths of MSC were 4.61, 3.87, 4.00, 4.04, 3.27, 3.28, and 3.13 MPa, respectively. Compared with the concrete without MS, the flexural strength reduction rates were 16.05%, 13.23%, 12.36%, 29.07%, 28.85%, and 32.10%, respectively. When the MS content is 0~60% for 28 d, the flexural strengths of MSC were 5.74, 5.06, 4.44, 4.32, 4.01, 3.95, and 3.94 MPa, respectively. Compared with the concrete without MS, the flexural strength reduction rates were 11.85%, 22.64%, 24.47%, 30.14%, 31.18%, and 31.35%, respectively. The results show that the flexural strength of concrete decreases greatly when the content of MS is between 0% to 40%. When the content of MS exceeds 40%, the flexural strength decreases gradually with the increase of MS content.

#### 3.2.2. Influence of Water-Cement Ratio on the Flexural Strength of MSC

**Figure 6:**
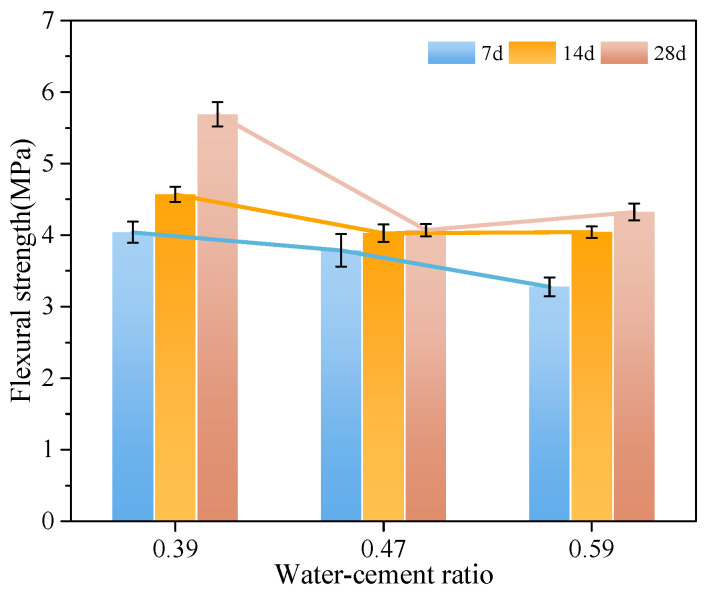
Flexural strength of carbon curing MSC with different water-cement ratio.

To study the influence of different water-cement ratios on the flexural strength of MSC, the flexural strength of MSC with curing age under water-cement ratios of 0.59, 0.47, and 0.39 was analyzed in this experiment. The flexural strength of MSC with different water-cement ratios is shown in Figure 6.

It is concluded from Figure 6 that with the increase of water-cement ratio, the flexural strength of MSC decreases in different degrees.

When the water-cement ratios are 0.39, 0.47, and 0.59 for 7 d, the flexural strengths of MSC were 4.04, 3.79, and 3.28 MPa, respectively. Compared with the MSC of 0.39 water-cement ratio, the reduction rates of the flexural strength were 6.19% and 18.81%. For 14 d, the flexural strengths of MSC were 4.57, 4.03, and 4.04 MPa, respectively. Compared with the MSC of 0.39 water-cement ratio, the reduction rates of the flexural strength were 11.82% and 11.60%. For 28 d, the flexural strengths of MSC were 5.76, 4.07, and 4.32 MPa, respectively. Compared with the MSC of 0.39 water-cement ratio, the reduction rates of the flexural strength were 29.34% and 25.01%.

### 3.3. Mechanical Model of Carbon Cured MSC

A comprehensive model of the mechanical properties of MSC is established by analyzing the rules and reasons of the mechanical properties.

The compressive strength of the MSC decreases with the increase of the MS content. The reason for the above phenomenon is that the particle size of MS is reduced after grinding, and the grading of concrete can be improved with less MS [33]. The micro-expansion effect of MS after hydration can inhibit the shrinkage and cracking behavior of concrete during curing, making the inside of concrete denser [34]. The carbon curing system can quickly convert the cementing material on the surface of concrete into calcium carbonate and active silica gel, which supports the early strength of concrete. These reasons jointly promote the compressive enhancement of carbon curing MSC. However, due to the poor activity of MS, the hydration activity of MS is lower than that of cement, so the compressive strength of concrete will be significantly reduced in large dosage. In addition, the expansion property of MS causes expansion cracking to the internal structure of concrete in large dosages. Therefore, the mechanical properties of concrete have an obvious decreasing trend under the condition of adding large amounts of MS.

In the process of hydration reaction, the reduction of water-cement ratio can reduce the number of holes produced when free water evaporates in the concrete, making the concrete interior denser. In the process of carbonation reaction, the reduction of the water-cement ratio reduces the moisture content in the concrete gel hole, making it easier for CO_2_ to enter the concrete interior to obtain more calcium carbonate and active silica gel. The water-cement ratio affects the hydration and carbonation reaction of MSC, and thus affects the mechanical properties of MSC.

Based on the above change rules, the mechanical properties of MSC is influenced by various factors. The influences of curing age, water-cement ratio, and MS content on the mechanical properties of MSC are also considered. Finally, comprehensive models for the compressive strength and flexural strength of concrete are established. As shown in Equations (2) and (3), the fitting error *R*^2^ of the models is 0.94 and 0.93, respectively.

(2) $$f_{c}=64.70\times (-0.78\times m^{2}-1.12\times {\left(W/C\right)}^{2}+0.004\times d+1)$$

(3) $$f=1.37\times (-0.36\times {\left(W/C\right)}^{2}+3.0125\times d^{2}-2.32\times m+1)$$

where *f* and *f_c_* are the flexural strength and compressive strength, respectively. *d* is the curing age, *W*/*C* is the water-cement ratio, *m* is the MS content.

It is worth noting that the established MSC compressive strength model is only applicable to the MS content of 0%~60%, and the water-cement ratio is between 0.39~0.59. In addition, the water absorption characteristics of MS and the potential expansion hazards of MgO in the slag may affect the accuracy of the model in predicting the mechanical properties of MSC. Although some samples have a certain dispersion phenomenon, the model curve takes into account the MS content, water-cement ratio, curing age, and other factors, which can fully reflect the mechanical properties of MSC. This model can provide references for engineering applications and finite element simulations.

### 3.4. The Relationship Between the Compressive Strength and the Flexural Strength of MSC

There is a correlation between the compressive strength and flexural strength of MSC. Affected by several factors, the specific functional relationship between the two parameters will be different. Under the same test piece size and preparation conditions, the flexural strength of MSC will be 1.5–2.0 times lower than the compressive strength. Because concrete is subjected to transverse shear forces as well as longitudinal forces, it exhibits low strength in flexural resistance.

**Figure 7:**
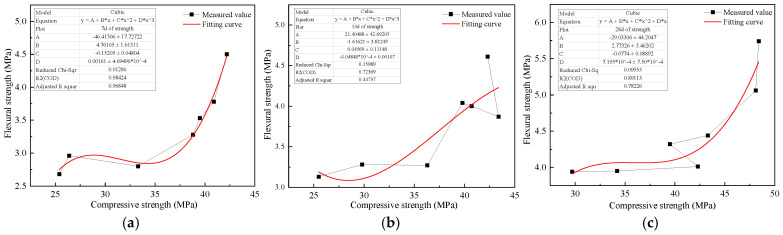
Relationship models of flexural and compressive strength. (**a**) 7 d, (**b**) 14 d, (**c**) 28 d.

Based on the above change rules, the flexural strength and compressive strength of MSC at different ages of 7 d, 14 d, and 28 d were fitted, and the mechanical relationship models of MSC were established. The models are shown in Figure 7, and model parameters are as Equation (4). Coefficients in Equation (4) are shown in Table 4.

(4) $$f=A{f_{c}}^{3}+B{f_{c}}^{2}+Cf_{c}+D$$

where, *f* is the flexural strength, and *f_c_* is the compressive strength of MSC.

The establishment of an MSC compression-flexural strength model will help to calculate the overall properties of materials by using small size samples, and promote the industrial application and popularization of MSC.

## 4. Microscopic Properties of MSC

The microscopic properties of CO_2_ cured MSC with different MS contents, curing ages, and water-cement ratios were studied. Through TGA, the curve of MSC quality changing with temperature was obtained, and the CSC of MSC was obtained. The phase change of MSC was determined by XRD and the hydration reaction of the MS—cement cementite system with different contents was analyzed.

### 4.1. Thermal Weight Analysis of MSC

**Figure 8:**
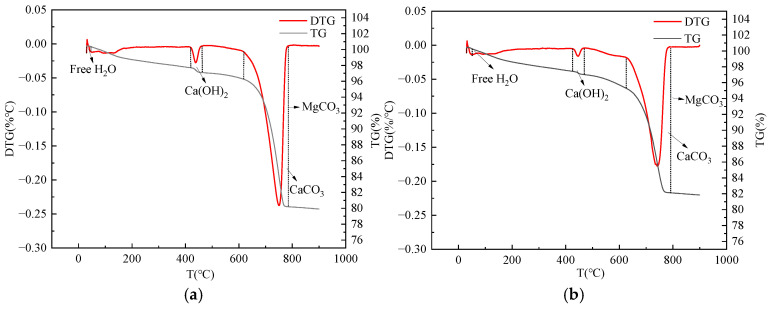
Thermal analysis image of MSC with 0% MS content. (**a**) 3 d, (**b**) 28 d.

**Figure 9:**
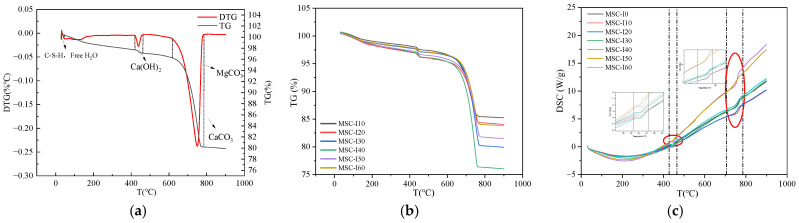
Thermal analysis image of MSC with different MS content at 28 d. (**a**) TG-DTG, (**b**) TG, (**c**) DSC.

The thermal weight of concrete is analyzed with different MS contents, which were 0%, 10%, 20%, 30%, 40%, 50%, and 60%. The thermal weight test results of MSC with curing age are shown in Table 5 and Table 6, and the change curve of the thermal weight of MSC with curing age is shown in Figure 8 and Figure 9.

Figure 8 displays the thermal analysis results for MSC after carbon curing, revealing three primary weight loss peaks. Peaks corresponding to free water, C-S-H gel, and Aft are observed around 100 °C. The weight loss at 28 d is lower than at 3 d, attributed to moisture consumption during subsequent hydration reactions. The second peak represents Ca(OH)_2_, which diminishes due to reaction with CO_2_ during carbonation curing. Ca(OH)_2_ content increases from 3 to 28 d as hydration progresses. The third peak results from CaCO_3_ and MgCO_3_ decomposition, providing early strength support for MSC. Figure 9a,b shows the 28 d TG-DTG curves of carbon cured MSC with different MS contents, and it can be seen in Figure 9a and Table 5 that the carbon fixation of MSC is about 10% to 20%. The higher the content of MS, the stronger the alkaline reserve and better the carbonation reaction is promoted. However, a MS content of more than 50% reduces the carbon sequestration of concrete. This is attributed to the fact that the dense surface layer prevents deep carbonation. Figure 9c shows the heat flow curve of the reaction, with two most obvious exothermic peaks. The first is a dehydration decomposition of Ca(OH)_2_ at 450 °C. The second peak is located at 600~900 °C, which is the endothermic decomposition peak of carbonate products (CaCO_3_, MgCO_3_). This is similar to the change law of mechanical properties, which proves that the increase of carbonation degree has an effect on the improvement of concrete strength, that is, more carbonation products play a filling role to compact the concrete matrix and improve the pore structure, thus effectively improving the strength of concrete.

Table 6 depicts the relationship between water-cement ratio and carbon fixation in carbon cured MSC. Reducing the water-cement ratio from 0.59 to 0.47 increases carbon sequestration by 1.9%. However, a further reduction to 0.39 results in a decrease in carbon sequestration to 10.18%. Excessive moisture will hinder the transfer of CO_2_ in the concrete matrix and inhibit the carbonation reaction, and the dense matrix will also hinder the diffusion of CO_2_ at a low water-cement ratio. Therefore, the selection of moderate water-cement ratio conditions is crucial to the control of the degree of carbonation.

The increase of concrete strength is caused by the double action of water-cement ratio and high carbonation degree.

### 4.2. X-Ray Diffraction Analysis of MSC

**Figure 10:**
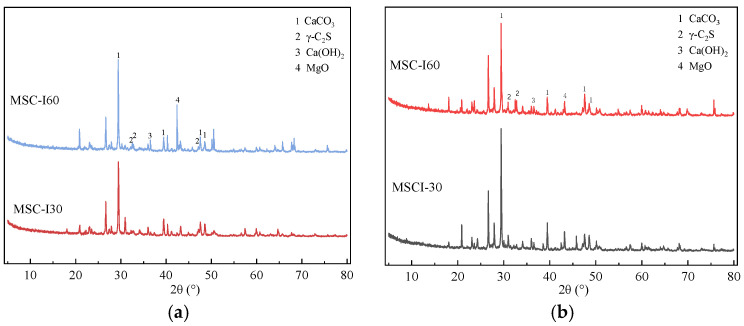
X-ray diffraction results of MSC with different MS content. (**a**) 3 d, (**b**) 28 d.

**Figure 11:**
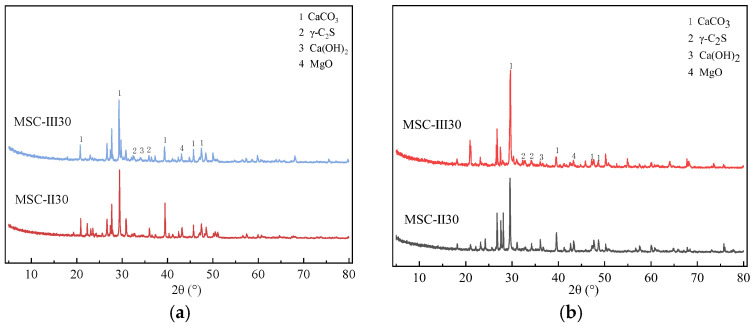
X-ray diffraction results of MSC with different water-cement ratios. (**a**) 3 d, (**b**) 28 d.

The X-ray diffraction analysis of MSC is analyzed with different MS contents at 3 d and 28 d, as shown in Figure 10 and Figure 11.

Figure 10 shows that the main phases of MSC consist of CaCO_3_, γ-C_2_S, Ca(OH)_2_, and MgO. The carbonation mechanism of MSC is similar to that of cement. With the increase of MS content, more CaCO_3_ with higher crystallinity will be formed in the early stage. But with the increase in age, the crystallinity and content of CaCO_3_ in the sample MSC-I30 with high degree of carbonation regain the advantage in the groups with different MS content, which shows higher and sharper peaks in the X-ray diffraction figure. This is consistent with the laws of thermogravimetric analysis and mechanical properties of concrete. The increase of MS content also increases the content of MgO in concrete. The presence of large contents of f-MgO are not good for concrete, affecting the bulk stability of concrete. In the hydration process of concrete, Ca(OH)_2_ is also produced. Due to the conversion of Ca(OH)_2_ to CaCO_3_ in carbonation curing, it shows a low peak on XRD. 

Figure 11 shows the XRD changes of MSC with different water-cement ratios during curing days 3 and 28. The peak shape of CaCO_3_ becomes sharper compared to that at 3 d, indicating that more low-crystallinity CaCO_3_ is produced at a lower water-cement ratio, which gradually converts to a high-crystallinity calcite form as the curing age increases. The formation of calcium carbonate is accompanied by the improvement of mechanical properties of MSC. This shows the importance of water-cement ratio on the mechanical properties of MSC.

## 5. Conclusions

The purpose of the paper was to investigate the variation of compressive and flexural strength of MSC under CO_2_. The carbonation reaction mechanism and carbon fixation change rule were analyzed based on thermal weight analysis and X-ray diffraction analysis.

(1) The incorporation of MS significantly affects the carbonation curing and mechanical properties of concrete. As the proportion of MS increases, both compressive strength and flexural strength decrease. The turning point for changes in compressive strength occurs at approximately 30% MS content, while for flexural strength it appears at around 40%. Regarding the resistance to flexural strength after 14 d, an increase is observed with the addition of 10% to 30% MS, with growth rates of 3.36% and 0.99%, respectively, reaching a peak at 30%. With the further increase of MS content, the effect on the concrete turns to deterioration, resulting in a significant reduction in compressive strength, which is about 19% after 28 d.
(2) The change of water-cement ratio on the strength of concrete is also very significant. When the water glue is relatively low, the proportion of water in the concrete is reduced, resulting in a reduction in the porosity of the concrete. In addition, it promotes the full hydration reaction of the cement, thereby improving the strength of the concrete. Therefore, with the increase of the water-cement ratio, the strength of the concrete gradually decreases. From the fitting model of the compressive strength of MSC, the amount of MS and water-cement ratio have more influence on the compressive strength than the curing age. For the flexural strength, the water-cement ratio and maintenance age have greater influence than that of MS.
(3) Appropriately increasing the amount of MS can improve the absorption of CO_2_. The content of MS increased from 0% to 60%, and the carbon sequestration content was 14.60%, 11.87%, 11.69%, 16.90%, 19.80%, 14.78%, and 13.09%, respectively. When the content of MS is 40%, the carbon fixation of concrete is 19.8%. As for the water-cement ratio, the carbon fixation of concrete increases first and then decreases with the increase of water-cement ratio. Reducing the water-cement ratio from 0.59 to 0.47 increases carbon sequestration by 1.9%. However, a further reduction to 0.39 results in a decrease in carbon sequestration to 10.18%.
(4) With the increase of MS content, CaCO_3_ in MSC increased. With the increase of concrete age, the CaCO_3_ content and the intensity of reflection peak in concrete also increase gradually. This is because the cement inside the concrete constantly hydrates to release the Ca(OH)_2_ with the CO_2_ in the air, which reacts to generate CaCO_3_, thereby meaning that the CaCO_3_ was gradually increased.

## Figures and Tables

**Table 1:** Physical and mechanical properties of cement.

Water Requirement of Normal Consistency/%	Specific Area/m^2^·kg^−1^	Stability	Loss on Ignition/%	Setting Time/h		Compressive Strength/MPa		Flexural Strength/MPa	
				Initial Set	Final Set	3 d	28 d	3 d	28 d
25.8	334	qualified	2.79	2.3	3.4	28.8	48.6	6.4	8.6

**Table 2:** Comparison of chemical composition of cement MS (%).

Composition/Content/%	CaO	SiO_2_	MgO	Fe_2_O_3_	Al_2_O_3_	SO_3_	MnO_2_	CuO	Other
Cement	63.42	21.18	5.02	3.14	5.02	2.3	-	-	1.82
MS	60.93	28.67	5.10	3.42	0.73	0.032	0.058	0.012	1.048

**Table 3:** Material dosage per cubic meter of MSC (kg).

Number	Cement	MS	Coarse Aggregate			Fine Aggregate	Water	Water Reducer
			5–10 mm	10–16 mm	>16 mm			
MSC-I0	366	0	581.5	523.3	58.1	683	161	3.66
MSC-I10	329.4	36.6	581.5	523.3	58.1	683	161	3.66
MSC-I20	292.8	73.2	581.5	523.3	58.1	683	161	3.66
MSC-I30	256.2	109.8	581.5	523.3	58.1	683	161	3.66
MSC-I40	219.6	146.4	581.5	523.3	58.1	683	161	3.66
MSC-I50	183	183	581.5	523.3	58.1	683	161	3.66
MSC-I60	146.4	219.6	581.5	523.3	58.1	683	161	3.66
MSC-II30	277.2	118.8	581.8	523.6	58.2	683.4	150.4	3.96
MSC-III30	338.1	144.9	513.1	461.8	51.3	774.1	140.0	4.83

**Table 4:** Mechanical model coefficients of MSC.

Age/Coefficients	A	B	C	D
7 d	0.0016	−0.15	−4.76	−46.42
14 d	−0.0004	−0.05	−1.62	21.4
28 d	0.0007	2.77	2.77	−29.03

**Table 5:** Carbon sequestration of MSC with different MS content at 28 d.

Number	MSC-I0	MSC-I10	MSC-I20	MSC-I30	MSC-I40	MSC-I50	MSC-I60
Carbon sequestration (%)	14.60	11.87	11.69	16.90	19.80	14.78	13.09

**Table 6:** Carbon sequestration of MSC with different water-cement ratios at 28 d.

Water-cement ratio	0.39	0.47	0.59
Carbon sequestration (%)	10.18	18.80	16.90

## Data Availability

Data sharing not applicable.

## References

[1] Amini O., Ghasemi M. (2019). Laboratory study of the effects of using magnesium slag on the geotechnical properties of cement stabilized soil. Constr. Build. Mater..

[2] Wang X., Yan X., Li X. (2020). Environmental risks for application of magnesium slag to soils in China. J. Integr. Agric..

[3] Pappu A., Saxena M., Asolekar S.R. (2007). Solid wastes generation in India and their recycling potential in building materials. Build. Environ..

[4] Courtial M., Cabrillac R., Duval R. (1991). Feasibility of the Manufacturing of Building Materials from Magnesium Slag.

[5] Pang Y., Zhang Z., Wang K., Wang C. (2022). Successful experience of calcining cement clinker with magnesium slag batching. Cement.

[6] Qiang Y., Zhou Y., Peng J., Han J., Cui J. (2020). Production practice of magnesia slag replacing limestone for clinker production. Cem. Eng..

[7] Li H., Huang Y., Yang X., Jiang Z., Yang Z. (2018). Approach to the management of magnesium slag via the production of Portland cement clinker. J. Mater. Cycles Waste Manag..

[8] Wen X., Zhang J., Liao H., Cheng F. (2019). Study on crystal phase transformation of fiber feedstock mixed with fly ash and magnesium slag during temperature rise. Compr. Util. Fly Ash.

[9] Gao L. (2021). Study on porosity control of porous ceramic filter balls made from magnesium slag. China Met. Bull..

[10] Ge T., Fan Y., Li H., Cheng F. (2015). Effects of magnesium slag silica-potassium fertilizer on salinity soil fertility and rape growth. Guangdong Agric. Sci..

[11] Zhang X. (2023). Application of metal magnesium slag in cement production. Cem. Technol..

[12] Xiao L., Wang S., Luo F. (2008). Application status and prospect analysis of industrial waste slag such as magnesium slag. J. Jilin Univ. Civ. Eng. Archit..

[13] Sun R., Wu Z., Wang D., Ding Y. (2023). Properties and hydration mechanism of ultrafine magnesium slag and cement composite cementing materials. Mater. Rev..

[14] Ruan Y., Pan G., Gao Q. (1997). Study on preparation of ultra-high strength concrete by ultrafine pulverized fly ash. J. Dalian Univ. Technol..

[15] Luo F. (2010). Research and Application of Magnesium Slag Gelling Materials with Less Clinker. Master’s Thesis.

[16] Peng X., Wang K., Li J., Yu Z. (2013). Activation of magnesium slag and preparation of Magnesium slag brick. J. Chongqing Univ. (Nat. Sci. Ed.).

[17] Wang Y., Zhang Z. (2011). Study on alkali activated magnesium slag cementing materials. China Pet. Chem. Stand. Qual..

[18] Lu G., Han J., Ma Z. (2022). Experimental design of gelling characteristics of alkali excited magnesium slag. Exp. Technol. Manag..

[19] Yang T., Zhang Z., Zhu H., Gao X., Dai C., Wu Q. (2019). Re-examining the suitability of high magnesium nickel slag as precursors for alkali-activated materials. Constr. Build. Mater..

[20] Meng L., Wang Z., Guo Z. (2022). Effective separation of fusing agent from refined magnesium slag by supergravity technology. Chem. Eng. Process.—Process Intensif..

[21] Zhou J., Cui Z., Zhou K., Zheng H. (2012). Study on the effect of magnesium slag replacing some fine aggregate on compressive strength of concrete. Concrete.

[22] Longarini N., Crespi P., Zucca M., Giordano N., Silvestro G.D. (2014). The Advantages of Fly Ash Use in Concrete Structures. J. Pol. Miner. Eng. Soc..

[23] Heng X., Lu J., Li X., Qing P., Xin C. (2013). Progress in Research of Effect of Silica Fume on the Performance of Cement Concrete. Bull Ceramic..

[24] Liu X., Wu P., Liu X., Zhang Z., Ai X. (2024). The Utilization of Carbonated Steel Slag as a Supplementary Cementitious Material in Cement. Materials.

[25] Zhang D., Ghouleh Z., Shao Y. (2017). Review on carbonation curing of cement-based materials. J. CO_2_ Util..

[26] Liu Z., Meng W. (2021). Fundamental understanding of carbonation curing and durability of carbonation-cured cement-based composites: A review. J. CO_2_ Util..

[27] Hao Y., Mo L., Yun J. (2016). Effect of carbonization on strength and microstructure of magnesium slag mortar. Bull. Silic..

[28] Ye J., Liu S., Zhao Y., Li Y., Fang J., Zhang H., Guan X. (2023). Development of Ultrafine Mineral Admixture from Magnesium Slag and Sequestration of CO_2_. Buildings.

[29] Mo L., Hao Y., Liu Y., Wang F., Deng M. (2019). Preparation of calcium carbonate binders via CO_2_ activation of magnesium slag. Cem. Concr. Res..

[30] Mo L., Zhang F., Panesar D.K., Deng M. (2017). Development of low-carbon cementitious materials via carbonating Portland cement–fly ash–magnesia blends under various curing scenarios: A comparative study. J. Clean. Prod..

[31] Dai Y., Ma J., Guo C., Xu X. (2020). Experimental Study on Carbonization of Magnesium Slag Cement. E3S Web Conf..

[32] Gao Y.-H., Liu L., Fang Z.-Y., He W. (2024). A backfill material without cementitious material: Carbonation curing magnesium slag based full solid waste backfill material. J. Cent. South Univ..

[33] Li Z. (2016). Research on Carbonization Characteristics of Magnesium Slag Concrete. Master’s Thesis.

[34] Cui Z., Li S., Zhang C., Chen D. (2015). Drying shrinkage characteristics of magnesium slag and fly ash composite concrete. Adv. Eng. Sci..

